# Estimation of Relative Bioavailability of Lead in Soil and Soil-Like Materials Using Young Swine

**DOI:** 10.1289/ehp.8852

**Published:** 2006-04-04

**Authors:** Stan W. Casteel, Christopher P. Weis, Gerry M. Henningsen, William J. Brattin

**Affiliations:** 1 Veterinary Medical Diagnostic Laboratory, College of Veterinary Medicine, University of Missouri, Columbia, Missouri, USA; 2 U.S. Environmental Protection Agency, National Enforcement Investigations Center, Denver, Colorado, USA; 3 H & H Scientific Services LLP, Evansville, Indiana, USA; 4 Syracuse Research Corporation, Denver, Colorado, USA

**Keywords:** lead, RBA, relative bioavailability, swine

## Abstract

In this article we summarize the results of a series of studies that measured the relative bioavailability (RBA) of lead in a variety of soil and soil-like test materials. Reference material (Pb acetate) or Pb-contaminated soils were administered orally to juvenile swine twice a day for 15 days. Blood samples were collected from each animal at multiple times during the course of the study, and samples of liver, kidney, and bone were collected at sacrifice. All samples were analyzed for Pb. We estimated the RBA of a test material by fitting mathematical models to the dose–response curves for each measurement end point and finding the ratio of doses that gave equal responses. The final RBA for a test material is the simple average of the four end point–specific RBA values. Results from 19 different test materials reveal a wide range of RBA values across different exposure materials, ranging from 6 to 105%. This variability in RBA between different samples highlights the importance of reliable RBA data to help improve risk assessments for Pb in soil. Although the RBA value for a sample depends on the relative amounts of the different chemical and physical forms of Pb present, data are not yet adequate to allow reliable quantitative predictions of RBA from chemical speciation data alone.

Reliable evaluation of the potential hazard to children from ingestion of lead in the environment depends in part on accurate information on the rate and extent of Pb absorption (“bioavailability”) from each exposure medium. This is especially true for soil because Pb in soil can exist in a variety of different mineral forms and particle types, some of which tend to have low absorbability. Thus, equal ingested doses of different forms of Pb in soil may not be of equal health concern.

Oral bioavailability of Pb in a particular medium may be expressed either in absolute terms [absolute bioavailability (ABA)] or in relative terms [relative bioavailability (RBA)]. ABA is the fraction of Pb that reaches the systemic circulation after oral ingestion. Typically, ABA is measured by comparing the time course of absorption after both oral and intravenous (iv) doses and comparing the area under the curve (AUC) of blood Pb concentration versus time:


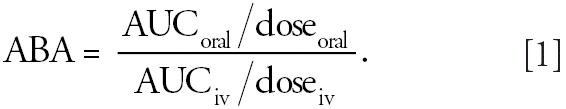


This ratio is also referred to as the oral absorption fraction. RBA is the ratio of the ABA of Pb present in some test material compared with the ABA of Pb in some appropriate reference material:


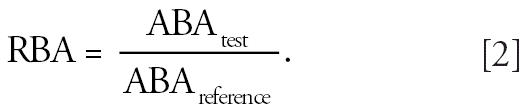


Usually, the form of Pb used as a reference material is a soluble compound, such as Pb acetate, that is expected to completely dissolve in gastrointestinal fluids when ingested.

We have been engaged in a multiyear investigation of Pb absorption in juvenile swine after oral exposure to a variety of different environmental media, especially soils and solid wastes associated with mining, milling, and smelting sites. Initial studies in the program (referred to as “Phase 0” and “Phase I”) were performed by R. Poppenga and B. Thacker at Michigan State University (Weis et al. 1995). The study designs and protocols developed during the early studies were refined and standardized (by S. Casteel, at the University of Missouri–Columbia) and applied to a number of different test materials collected from various Superfund sites. This series of measurements is collectively referred to as “Phase II,” and the results are presented in this article. A more detailed presentation of the Phase II work, including raw data from all studies, is available from the U.S. Environmental Protection Agency (U.S. EPA 2006). Drexler and Brattin (in press) have compared the results of the Phase II *in vivo* studies with the results of an *in vitro* technique for estimating Pb RBA in soil samples.

## Materials and Methods

### Animals

Juvenile swine were selected for use in this program because they are considered a good model for the gastrointestinal system of a human child (U.S. EPA 2006; Weis et al. 1995). All animals were intact males of the Pig Improvement Corporation genetically defined Line 26, purchased from Chinn Farms (Clarence, MO). Animals were usually purchased at 4–5 weeks of age (weaning occurs at 3 weeks of age). In general, about 10% more pigs were purchased than were required for the experimental design. All animals were held under quarantine for 1 week to allow us to observe their health and cull any sick animals from the study. In addition, to minimize weight variations among animals and groups, we excluded extra animals that were most different in body weight (either heavier or lighter than average) 4 days before exposure began. The remaining animals were assigned to dose groups at random (typically five animals per group). When exposure began (day 0), the animals were about 5–6 weeks of age and weighed an average of approximately 8–11 kg.

All animals were housed in individual stainless steel cages. Each animal was examined by a certified veterinary clinician (swine specialist) before being placed on study, and each was examined daily by an attending veterinarian while on study. Blood samples were collected by venipuncture for clinical chemistry and hematologic analysis on days 4, 7, and 15 to assist in clinical health assessments. Any animal that became ill and could not be promptly restored to good health by appropriate treatment was removed from the study. All animals were treated humanely and with regard for alleviation of suffering.

### Diet

Animals provided by the supplier were weaned onto standard pig chow purchased from MFA Inc. (Columbia, MO). To minimize Pb exposure from the diet, the animals were gradually transitioned from the MFA feed to a special low-Pb feed (guaranteed < 0.2 mg/kg Pb; Zeigler Brothers Inc., Gardners, PA) from day −7 (7 days before exposure began) to day −3; this low-Pb feed was then provided for the duration of the study. The feed was nutritionally complete and met all requirements of the National Institutes of Health–National Research Council (NRC) for swine rations (NRC 1988). Periodic analysis of feed samples during this program indicated the mean Pb level was below the detection limit (0.05 mg/kg), corresponding to a daily intake of < 2.5 μg/kg/day.

Each day every animal was given an amount of feed equal to 5% of the mean body weight of all animals on study. Feed was administered in two equal portions of 2.5% of the mean body weight at each feeding. Feed was provided at 1100 hr and 1700 hr daily. Drinking water was provided *ad libitum* via self-activated watering nozzles within each cage. Periodic analysis of samples from randomly selected drinking water nozzles indicated the mean Pb concentration in water was < 2 μg/L, corresponding to a daily intake of < 0.2 μg/kg/day.

### Test materials

Table 1 describes the Phase II test materials for which RBA was measured and provides the analytical results for Pb. We investigated 17 different samples from eight different sites, along with one sample of paint flakes mixed with clean soil and one sample of finely ground native galena mixed with clean soil. Before analysis and dosing, all samples were dried (< 40°C) and sieved; only materials that passed through a 60-mesh screen (corresponding to particles smaller than approximately 250 μm) were used, with the exception of the two samples from study 5 (test materials 7 and 8), which were sieved to 150 μm. We selected this range of particle sizes because the U.S. EPA considers particles < 250 μm to be the most likely to adhere to hands and be ingested by children (U.S. EPA 2000b).

Each sample of test material that was evaluated in the swine bioassay program was thoroughly characterized with regard to mineral phase, particle size distribution, and matrix association using electron microprobe analysis. The relative Pb mass (RLM) in each phase is the length-weighted fraction of the total Pb in a sample that is present in a particular phase *i*, calculated by summing across all particles in phase *i* as follows:


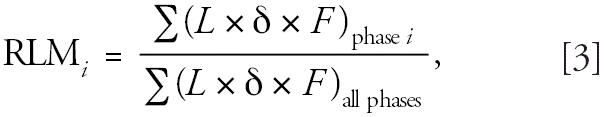


where RLM*_i_* is the RLM for phase *i*, *L* is the longest dimension of the particle, δ is the density of the particle, and *F* is the fraction (by mass) of Pb in the particle.

### Dosing

A typical study consisted of 10 dose groups. Dose group 1 usually consisted of three or five animals that were not exposed to any exogenous Pb (control group); all other dose groups consisted of five animals per group. Dose groups 2, 3, and 4 were exposed to Pb acetate, usually at doses of 25, 75, or 225 μg/kg/day. These dose levels were based on results from Phase 0 and Phase I investigations, which indicated that doses of Pb acetate in the range of 25–225 μg/kg/day Pb gave clear and measurable increases in Pb levels in all end points measured (blood, liver, kidney, bone). Animals in dose groups 5, 6, and 7 were exposed to test material 1, and animals in dose groups 8, 9, and 10 were exposed to test material 2. The doses of test materials were usually set somewhat higher than for Pb acetate (e.g., 75, 225, and 675 μg/kg/day Pb) so that measurable responses would still be likely even if the test material had a relatively low RBA. Depending on the concentration of Pb in the test material and the target dose level for Pb, soil intake rates needed to achieve target Pb doses were usually in the range of 0.5–2.5 g/day.

Animals were exposed to Pb acetate or test material for 15 days, with the dose for each day being administered in two equal portions given at 0900 hr and 1500 hr (2 hr before feeding). We selected these exposure times so that Pb ingestion would occur at a time when the stomach was largely or entirely empty of food, because the presence of food in the stomach is known to reduce Pb absorption (e.g., Blake et al. 1983; Chamberlain et al. 1978; Heard and Chamberlain 1982; James et al. 1985; Rabinowitz et al. 1980).

Dose material (Pb acetate or test material) was placed in the center of a small portion (~ 5 g) of moistened feed. This “doughball” was administered to the animals by hand. Dose calculations were based on measured group mean body weights and were adjusted every 3 days to account for animal growth. In most cases, the animals readily ingested the doughball, but occasionally an animal refused or dropped the dose. In this event, the date and amount of the missed dose were recorded and the time-weighted average dose calculation for each animal was adjusted downward accordingly.

### Sample collection and analysis

Samples of blood were collected from each animal 3 or 4 days before exposure began, on the first day of exposure (day 0), and on multiple days thereafter (usually days 1, 2, 3, 5, 7, 9, 12, and 15). All blood samples were collected by venipuncture of the anterior vena cava, placed immediately in purple-top Vacutainer tubes (Becton, Dickinson and Company, Franklin Lakes, NJ) containing calcium-EDTA (ethyl-enediamine tetra-acetic acid) as anticoagulant, and stored under refrigeration until analysis. Blood samples were collected each sampling day beginning at 0800 hr, approximately 1 hr before the first of the two daily exposures to Pb on the sampling day and 17 hr after the last Pb exposure the previous day. This blood collection time was selected because the rate of change in blood Pb resulting from the preceding exposures is expected to be relatively small after this interval (LaVelle et al. 1991; Weis et al. 1993).

One milliliter of whole blood from the purple-top Vacutainer was added to 9.0 mL of “matrix modifier” [0.2% (vol/vol) ultra-pure nitric acid, 0.5% (vol/vol) Triton X-100, and 0.2% (wt/vol; 0.015 M) dibasic ammonium phosphate in deionized, double-distilled water], a solution recommended by the Centers for Disease Control and Prevention (CDC) for analysis of blood samples for Pb (CDC 2001). Samples of the matrix modifier were routinely analyzed for Pb to ensure the absence of Pb contamination.

After collection of the final blood sample at 0800 hr on day 15, all animals were humanely euthanized, and samples of liver (medial lobe), kidney (both sides), and bone (the right femur) were removed and stored frozen in plastic bags for Pb analysis.

One gram of soft tissue (liver or kidney) was placed in a screw-cap Teflon container with 2 mL Optima-grade concentrated (70%) nitric acid and heated in an oven to 90°C overnight. After cooling, the digestate was transferred to a clean 10 mL volumetric flask and diluted to volume with deionized, double-distilled water.

The right femur of each animal was defleshed and dried at 100°C overnight. The dried bones were then broken in half, placed in a muffle furnace, and dry ashed at 450°C for 48 hr. After dry ashing, the bone was ground to a fine powder using a mortar and pestle, and 200 mg was removed and dissolved in 10.0 mL of 1:1 (vol:vol) Optima-grade concentrated nitric acid/water. After the powdered bone was dissolved and mixed, 1.0 mL of the acid solution was removed and diluted to 10.0 mL by addition of 0.1% (wt/vol) lanthanum oxide in deionized, double-distilled water.

Samples of biological tissue (blood, liver, kidney, bone) and other materials (e.g., food, water, reagents, solutions) were arranged in a random sequence and provided to the U.S. EPA analytical laboratory in a blind fashion (identified to the laboratory only by a chain-of-custody tag number). Each sample was analyzed for Pb using a PerkinElmer model 5100 graphite furnace atomic absorption spectrophotometer (PerkinElmer, Wellesley, MA). Internal quality control samples were run every 10th sample, and the instrument was recalibrated every 15th sample. A blank, duplicate, and spiked sample were run every 20th sample.

All results from the analytical laboratory were reported in units of micrograms of Pb per liter of prepared sample. The detection limit was defined as three times the SD of a set of seven replicates of a low-Pb sample (typically ~ 2–5 μg/L). The SD was usually about 0.3 μg/L, so the detection limit was usually about 0.9–1.0 μg/L. However, because different dilution factors were used for different sample types, the detection limit varied from sample type to sample type. For prepared blood samples (diluted 1/10), this corresponded to a detection limit of 10 μg/L (1 μg/dL). For soft tissues (liver and kidney, also diluted 1/10), this corresponded to a detection limit of 10 μg/kg wet weight. For bone (final dilution of 1/500), the corresponding detection limit was 0.5 μg/g ashed weight.

### Quality assurance

We took a number of steps throughout each of the studies to assess and document the quality of the data that were collected. These steps are summarized below.

#### Duplicates

We submitted a randomly selected set of about 5% of all blood and tissue samples generated during each study to the laboratory in a blind fashion for duplicate analysis. There was good reproducibility between duplicate samples for both blood and tissues, with both linear regression lines having a slope near 1.0, an intercept near zero, and an *R*^2^ value near 1.00.

#### Performance standards for blood

We obtained three sets of performance evaluation blood samples from the CDC, with nominal concentrations of 1.7 μg/dL, 4.8 μg/dL, and 14.9 μg/dL. Each day that blood samples were collected from experimental animals, several performance evaluation samples of different concentrations were also prepared and submitted for analysis in random order and in a blind fashion. Analytical results obtained for the performance evaluation samples were generally in good agreement with the expected value at all three concentrations, with an overall mean of 1.4 μg/L for the low standards (nominal concentration of 1.7 μg/L), 4.3 μg/L for the middle standards (nominal concentration of 4.8 μg/L), and 14.5 μg/L for the high standards (nominal concentration of 14.9 μg/L).

#### Interlaboratory comparison

In each study, we performed an interlaboratory comparison of blood Pb analytical results by sending a set of about 15–20 randomly selected whole-blood samples to the CDC for blind independent preparation and analysis. The results from the U.S. EPA laboratory were generally similar to those of the CDC, with a mean intersample difference (U.S. EPA value minus CDC value) of 0.07 μg/dL. The slope of the best-fit straight line through the paired data was 0.84, indicating that the concentration values estimated by the U.S. EPA laboratory tended to be about 15% lower than those estimated by the CDC. The reason for this apparent discrepancy between the U.S. EPA laboratory and the CDC laboratory is not clear but might be related to differences in sample preparation techniques. Regardless of the reason, the differences are sufficiently small that they are likely to have no significant effect on calculated RBA values. In particular, it is important to realize that if both the Pb acetate and test material dose–response curves are biased by the same factor, then the biases cancel in the calculation of the ratio.

### Approach for estimating RBA

The method we used to estimate the RBA of Pb in a particular test material compared with the reference material (Pb acetate) is based on the principle that equal absorbed doses of Pb will produce equal biological responses. By definition,


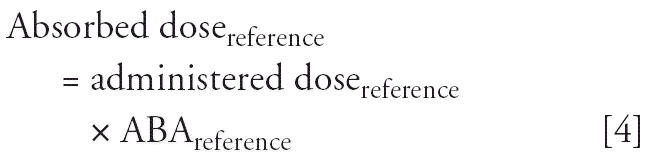


and


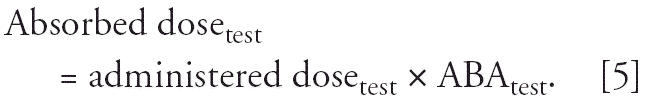


When responses are equal, then absorbed doses are equal, and


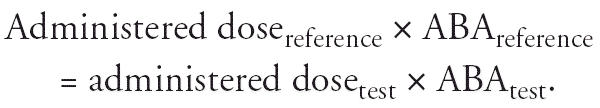


Thus,


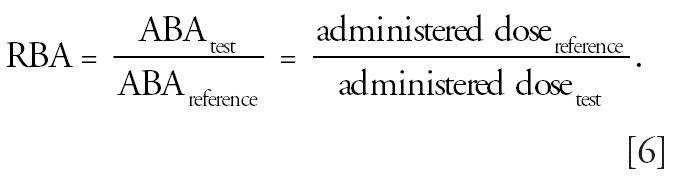


That is, given the dose–response curve for some particular end point (e.g., blood Pb AUC or the concentration of Pb in liver, kidney, or bone) for both the reference material and the test material, the RBA may be calculated as the ratio of administered doses that produce equal biological responses (and not as the ratio of responses at equal doses). In this approach, the mathematical form of the dose–response model must be the same for both reference material and test material. This is because the shape of the dose–response curve is a function only of the pharmacokinetic response of the biological organism to an absorbed dose of Pb, and the response per unit absorbed dose does not depend on whether the absorbed Pb was derived from reference material or test material.

### Statistical methods for fitting dose–response models

The techniques we used to derive statistical models of the dose–response data and to estimate the RBA are based on the methods recommended by Finney (1978). All model fitting was performed using JMP (version 3.2.2; SAS Institute Inc., Cary, NC).

As noted by Finney (1978), when the data to be analyzed consist of two or more dose–response curves from the same study (e.g., Pb acetate, test material 1, test material 2), it is apparent that all curves must have the same intercept, because there is no difference between the curves when the dose is zero. This requirement is achieved by fitting all of the data from a study simultaneously and requiring the intercept to be identical for each curve.

Regression analysis based on ordinary least-squares minimization assumes that the variance of the responses is independent of the dose and/or the response (Draper and Smith 1998). In the present studies, this assumption is generally not satisfied because variability in response tends to increase as a function of increasing dose (heteroskedasticity). One method for dealing with heteroskedasticity is through the use of weighted least-squares regression (Draper and Smith 1998). In this approach, each observation in a group of animals is assigned a weight that is inversely proportional to the variance of the response in that group:


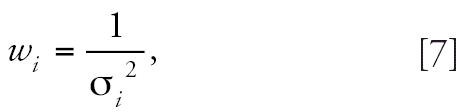


where *w_i_* is the weight assigned to all data points in dose group *i* and σ*_i_*^2^ is the variance of responses in animals in dose group *i*. We considered several options for estimating the value of σ*_i_*^2^:

- Option 1: using the observed variance (*s_i_*^2^) in the responses of animals in dose group *i*
- Option 2: establishing a variance model of the form σ*_i_*^2^ = αμ*_i_*^ρ^, where μ*_i_* is the predicted mean response for dose group *i*, and simultaneously fitting the data to derive values of α and ρ along with the other coefficients of the dose–response model using the data from a particular study—an approach identical to the nonconstant variance approach used by U.S. EPA Benchmark Dose Software (U.S. EPA 1995, 2001)
- Option 3a: establishing an “external” variance model based on an analysis of the relationship between variance and mean response using observations combined from all studies and dose groups, and using that model to predict the expected variance in dose group *i* as a function of the predicted mean response (i.e., the mean response predicted from the best-fit equation through the dose–response data) for that dose group
- Option 3b: establishing an “external” variance model based on an analysis of the relationship between variance and mean response using observations combined from all studies and dose groups, and using that model to predict the expected variance in dose group *i* as a function of the observed mean response level (i.e., the mean response measured in the exposed animals) for that dose group.

Based on a consideration of the advantages and disadvantages of each approach, we selected option 3b for use in this project; option 3b is relatively less vulnerable than other options to random variations in observed variances in a dose group (which results in assignment of weights that are either too high or too low). We preferred this option over option 3a because option 3a is based on predicted mean response, whereas option 3b is based on observed mean response. It should be noted, however, that option 3b is somewhat vulnerable to poor fits when one particular dose group in a data set lies well below the expected smooth fit through the other dose groups. In this case, the variance assigned to the group (based on the observed mean response) is lower than typical for that dose level (and hence the weights assigned to the data are higher than usual), tending to force the line through that data set at the expense of the other data sets.

**Figure 1:**
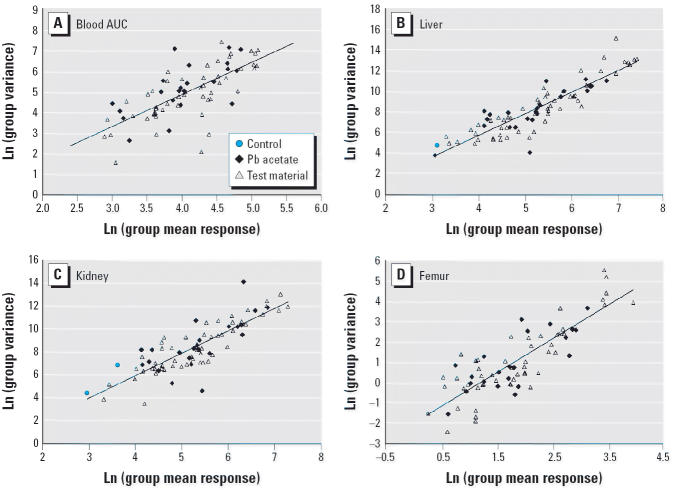
External variance models for (*A*) blood AUC (*y* = 1.5516*x* − 1.3226; *R*^2^ = 0.5046; *p* < 0.01); (*B*) liver (*y* = 2.0999*x* − 2.6015; *R*^2^ = 0.7966; *p* < 0.01); (*C*) kidney (*y* = 1.9557*x* − 1.8499; *R*^2^ = 0.7035; *p* < 0.01); and (*D*) femur (*y* = 1.656*x* − 1.9713; *R*^2^ = 0.7022; *p* < 0.01).

The external variance model for option 3b was based on the consolidated data from all studies (Phase II). In this analysis, some dose groups were excluded if the estimate of variance and/or mean response was judged to be unreliable, based on the following two criteria: *a*) the number of animals in the dose group was < 3, or *b*) the fraction of responses below the detection limit was > 20%. Figure 1 shows the log-variance in response plotted as a function of the log-mean response in the group for each of the end points. Log-variance increases as an approximately linear function of log-mean response for all four end points:


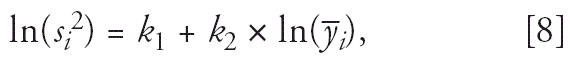


where 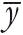 is the mean observed response of animals in dose group *i*.

Values of *k*_1_ and *k*_2_ were derived from the data for each end point using ordinary least-squares minimization. The resulting values are shown in Table 2. On the basis of these variance models, we assigned the weights for each response in a dose group based on the observed mean response for that dose group:


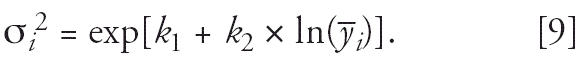


### Choice of model forms

As noted above, the main objective of the curve-fitting effort is to find a mathematical model that fits both the reference and test group dose–response data sets smoothly. There is no requirement that the model have a mechanistic basis or that the coefficients have a biological meaning. As discussed by Finney (1978), it is generally not appropriate to choose the form of the dose–response model based on only one experiment; the choice should be based on the weight of observations across many different studies. We evaluated four different models:

Linear:


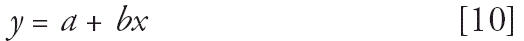



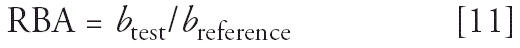


Exponential:


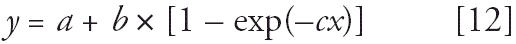



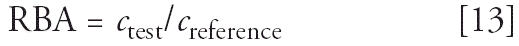


Michaelis-Menton:


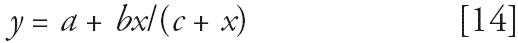



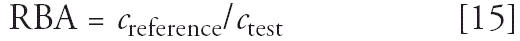


Power:


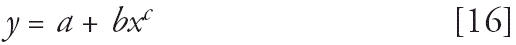



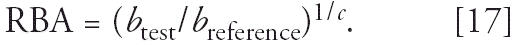


For each data set, the preferred model was identified based on Akaike’s information criterion (AIC) (U.S. EPA 2000a, 2001). On the basis of fitting each dose–response data set to each of the four models above, we found that the linear model most frequently gave the best fit for liver, kidney, and bone. In the few cases where the linear model was not the best fit, the RBA value given by the linear model was usually close to that given by whatever other model did provide the best fit. On this basis, we selected the linear model for application to all dose–response data sets for liver, kidney, and bone.

For the blood Pb AUC end point, the linear model usually gave the worst fit; therefore, we rejected it for the AUC end point. In general, each of the three nonlinear models (exponential, Michaelis-Menton, and power) tended to give similar results in terms of RBA value (the SD in RBA for a particular test material averaged across the three models was usually < 3%), and differences in the AIC were usually small. On this basis, we concluded that any of these three models would be acceptable. The power model was not selected because it does not tend toward a plateau, whereas data from early blood Pb pilot studies (using higher doses than commonly used in the Phase II studies) suggested that the blood Pb end point does tend to do so. Of the remaining two models (exponential and Michaelis-Menton), the exponential model was selected mainly because it yielded the best fit more often than did the Michaelis-Menton model and because the exponential model had been used in previous analyses of the data. Thus, the exponential model was selected for application to all dose–response data sets for the blood AUC end point, except in one special case, which is noted below.

In study 7 (test materials 11 and 12), the blood Pb AUC data set did not yield a solution in JMP for the exponential model, probably because the data have relatively less curvature than do most blood Pb AUC data sets. Because of this lack of curvature, it was not possible to estimate the exponential plateau value (*b*) with confidence, which in turn made it difficult to estimate the other parameters of the exponential model. Several alternative approaches for data reduction were evaluated, including using the model fits from one of the other nonlinear models, using the fit for the linear model, and fitting the data to the exponential model using a defined value for the plateau based on results from other data sets. The results (i.e., the RBA values based on the blood Pb AUC end point) were generally similar for all three of these approaches, so the results from the linear fit were used.

### Assessment of outliers

For the purposes of this program, end point responses that yielded standardized weighted residuals > 3.5 or less than −3.5 were considered to be potential outliers (Canavos 1984). A total of 13 such cases occurred out of a total of 1,895 end point responses (0.7%). In these cases, we calculated RBA values both with and without the outliers. In most cases, there was very little difference (the average ratio of RBA with outlier excluded to RBA with outlier included was 1.09). All results presented here are based on the analysis with outliers excluded.

### Uncertainty bounds in end-point–specific RBA values

The uncertainty bounds around each end-point–specific RBA value were estimated based on Fieller’s theorem, as described by Finney (1978).

### Combination of RBA estimates across end points

As discussed above, each study of RBA used four different end points to estimate absorption of Pb, including blood AUC, liver, kidney, and bone. Consequently, each study yielded four independent end-point–specific estimates of RBA for each test material. Thus, the final RBA estimate for a test material involves combining the four end-point–specific RBA values into a single value (point estimate) and estimating the uncertainty around that point estimate. The basic strategy selected for deriving a point estimate of RBA for a test material was to calculate a confidence-weighted average of the four end-point–specific RBA values. Because each end-point–specific RBA value is calculated as the ratio of the parameters of the dose–response curves fitted to the experimental data for reference material and test material, the relative confidence in an endpoint–specific RBA is inherently related to the quality of the data that define the dose–response curve for that end point. Thus, the indicator we selected to quantify the relative reliability of the four different end points is the magnitude of the uncertainty (SE) around RBA estimates based on each end point.

**Figure 2:**
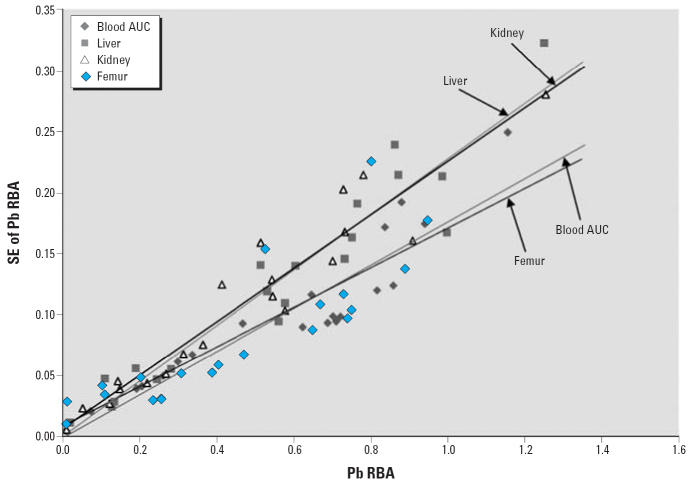
Evaluation of relative precision of measurement end points. *F*_crit_, critical frequency. For blood AUC, slope = 0.177, intercept = −0.002, and *R*^2^ = 0.867; for liver, slope = 0.227, intercept = 0.000, and *R*^2^ = 0.916; for kidney, slope = 0.219, intercept = 0.006, and *R*^2^ = 0.914; and for femur, slope = 0.162, intercept = 0.008, and *R*^2^ = 0.732. The results of the comparison of regression lines showed the following: *F* = 0.638; *F*_crit_(0.05) = 2.227; and *p* = 0.699.

Figure 2 shows the SE in each RBA estimate plotted as a function of the RBA value for each of the four different end points. Uncertainty in RBA (as reflected in the magnitude of the SE) increases as a function of the estimated value of RBA for all four end points. This is expected because of the heteroskedasticity in the underlying dose–response data. Although RBA values based on blood AUC or femur tend to yield estimates with slightly lower SEs than RBA values based on liver or kidney, the magnitude of the SEs tends to be generally similar for all four end points, and the difference between the four regression lines is not statistically significant (*p* = 0.699). Based on this, we judged each end-point–specific RBA value to have approximately equal validity; thus, we calculated the point estimate as the simple average across all four end-point–specific RBA values.

The uncertainty bounds around each point estimate were estimated using Monte Carlo simulation. Each end-point–specific RBA uncertainty distribution was assumed to be normal, with the mean equal to the best estimate of RBA and the SE estimated from Fieller’s theorem. In the Monte Carlo simulation, a value was drawn from one of the four uncertainty distributions, with an equal probability of choosing each of the distributions. The uncertainty in the point estimate was characterized as the range from the 5th to the 95th percentile of these random values.

## Results

### Dosing effects on animal health and weight

The Pb dose levels we used in this program were substantially below levels that cause clinical symptoms in swine, and we observed no evidence of treatment-related toxicity in any dose group. All animals exposed to Pb by the oral route remained in good health throughout each study; the only clinical signs observed were characteristic of normal swine. Animals typically gained about 0.3–0.5 kg/day, and the rate of weight gain was normally comparable in all exposure groups.

### Time course of blood Pb response

**Figure 3:**
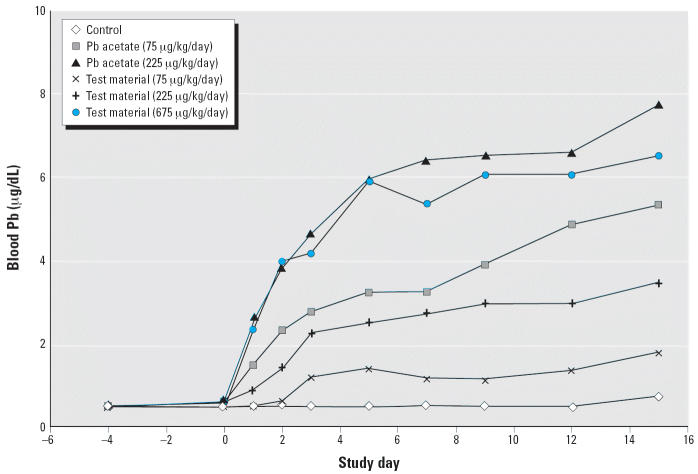
Example time course of blood Pb response to Pb acetate and test material.

Figure 3 presents an example graph of the time course of pseudo-steady-state blood Pb levels after repeated oral exposure to Pb acetate. Blood Pb levels began below the quantitation limit (usually ~ 1 μg/dL) and stayed very low in control animals throughout the course of the study. In animals exposed to Pb acetate, blood Pb values began to rise within 1–2 days and tended to flatten out to a near steady state within about 7–10 days. The temporal pattern was similar for test materials that were absorbed well enough to provide a clear response.

### Dose–response patterns

**Figure 4:**
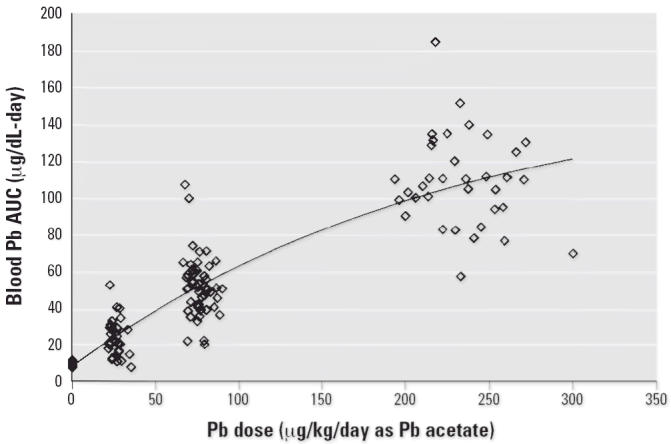
Dose–response curve for blood Pb AUC.

**Figure 5:**
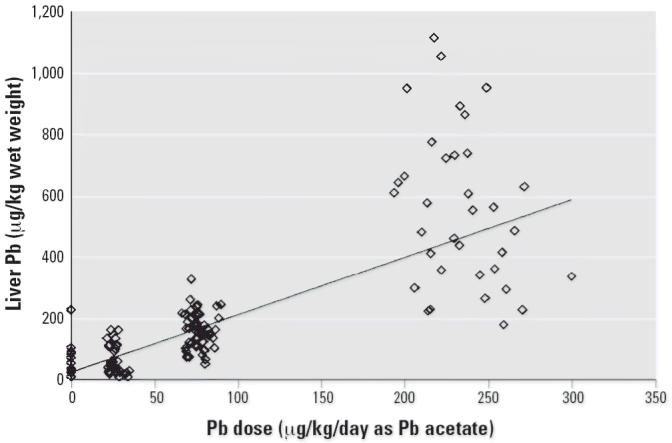
Dose–response curve for liver Pb concentration.

**Figure 6:**
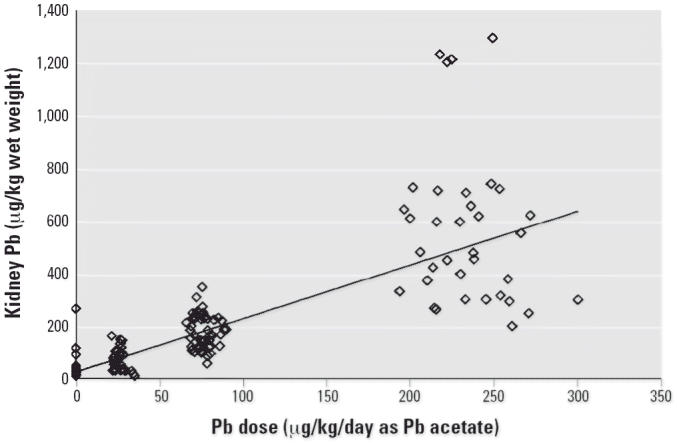
Dose–response curve for kidney Pb concentration.

**Figure 7:**
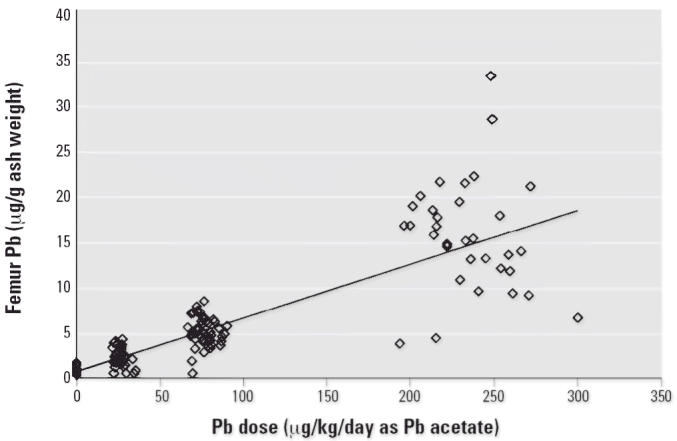
Dose–response curve for femur Pb concentration.

Figures 4–7 present the dose–response patterns observed for blood, liver, kidney, and bone (femur) after repeated oral exposure to Pb acetate. For blood, the end point is the blood Pb versus time AUC. For femur, kidney, and liver, the end point is the concentration in the tissue at the time of sacrifice. The data are based on the combined results across all studies performed during Phase II.

There was substantial variability in response between individuals (both within and between studies), and this variability tended to increase as dose (and response) increased. As noted above, this pattern of increasing variance in response (heteroskedasticity) is accounted for in the model-fitting procedure through the use of weighted least-squares regression. Despite the variability in response, it is apparent that the dose–response pattern was typically nonlinear for blood Pb AUC but was approximately linear for liver, kidney, and bone Pb. This pattern of dose–response relationships suggests that, at least over the dose range tested in this program, absorption of Pb from the gastrointestinal tract of swine is linear and that the nonlinearity observed in blood Pb AUC response was due to saturable binding in the blood compartment. This conclusion is based on the logic that, if the nonlinear behavior observed for blood were due to nonlinear absorption from the gastrointestinal tract, it would be extremely unlikely that all three of the other end points observed (liver, kidney, bone) would respond linearly.

### Characterization of test materials

Table 3 lists the different Pb phases observed in the test materials. Only a few of the phases are stoichiometric minerals (anglesite = Pb sulfate, cerussite = Pb carbonate, galena = Pb sulfide, native Pb = Pb), whereas the others are non-stoichiometric associations of various metals and other elements. As shown in Table 3, of the 22 different phases observed in one or more samples, nine are very minor, with RLM values no higher than 2% in any sample. However, 13 of the phases occur at concentrations that could contribute significantly to the overall bioavailability of the sample (RLM > 10%). It should be noted that a Pb-bearing particle that is present in a bulk sample from a slag pile is classified as slag only if the particle is glassy or vitreous in nature. Inclusions or other nonvitreous grains of Pb-bearing material that may be present are classified according to their mineral content [e.g., Pb oxide (PbO), galena].

Table 4 summarizes information on the degree to which Pb-bearing particles in each sample are partially or entirely liberated (i.e., exposed to gastric fluids when ingested) or included (i.e., fully enclosed or encased in mineral or vitreous matrices). Data are presented both on a particle frequency basis and on the basis of RLM. The majority of Pb-bearing particles in most samples were partially or entirely liberated, although test material 19 (Oregon Gulch tailings) is a clear exception. Table 5 summarizes data on the frequency distribution of particle sizes (measured as the longest dimension) in each sieved sample. For convenience, the data presented are for liberated particles only. Most samples contained a range of particle sizes, often with the majority of the particles being < 50 μm long.

### RBA results for test materials

End-point–specific RBA estimates for each test material are summarized in Table 6. Inspection of the final point estimates for the different test materials reveals a wide range of values across different samples, both within and across sites. For example, at the California Gulch site in Colorado, RBA estimates for different types of material range from about 6% (test material 19, Oregon Gulch tailings) to about 105% [test material 12, Fe/Mn (iron/manganese) PbO sample]. This wide variability highlights the importance of obtaining and applying reliable RBA data to site-specific samples in order to help improve risk assessments and more efficiently focus risk management of childhood Pb exposure.

### Reproducibility

Only one sample (test material 14, Palmerton location 2) was analyzed in duplicate during the Phase II study. As shown in Table 6, agreement is moderately good between the two studies for the blood AUC and kidney end points and for the point estimate, although there is relatively low agreement for the liver and bone end points.

### Correlation of RBA with mineral phase

In principle, each unique combination of phase, size, and matrix association constitutes a unique mineralogic form of Pb, and each unique form could be associated with a unique RBA that is the inherent value for that “type” of Pb. If so, then the expected RBA value observed for a sample containing a mixture of different “types” of Pb is the concentration-weighted average across all of the unique forms present in the sample. If the number of different Pb phases that may exist in the environment is on the order of ≥ 20, the number of size categories is on the order of five, and the number of matrix association categories is two (included, liberated), then the total number of different “types” of Pb is on the order of ≥ 200. Because measured RBA data are available from this study for only 19 different samples, it is clearly impossible with the present data set to estimate “type-specific” RBA values for each combination of phase, size, and matrix association. Therefore, to simplify the analysis process, we assumed that the measured RBA value for a sample was dominated by the liberated mineral phases present, and we did not consider the effects of included materials and particle size. That is, the data were analyzed according to the following model:


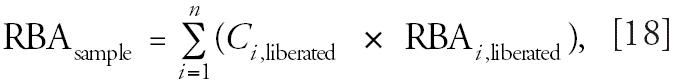


where RBA_sample_ is the observed RBA of Pb in a sample, *C_i_*_, liberated_ is the fraction of total Pb in liberated particles of phase *i*, *RBA_i_*_, liberated_ is the RBA of Pb in liberated particles of phase *i*, and *n* is the number of different Pb phase categories.

Because 22 different phases were identified and only 19 different samples were analyzed, it was necessary to reduce the number of phases to a smaller number so that regression analysis could be performed. Therefore, the different phases were grouped into 10 categories, as shown in Table 7. These groups were based on professional judgment regarding the expected degree of similarity among the different phases, along with information on the relative abundance of each phase (Table 3). The total Pb mass in each phase grouping was calculated by summing the RLM for each individual component in the group. As noted above, only the Pb mass in partially or entirely liberated particles was included in the sum. Group-specific RBA values were estimated by fitting the grouped data to the model using minimization of squared errors. Each parameter was constrained to be ≥ 0. Because group 10 contains only phases that are present in relatively low levels, an arbitrary coefficient of 0.5 was assumed for this group, and the coefficient was not treated as a fitting parameter.

**Figure 8:**
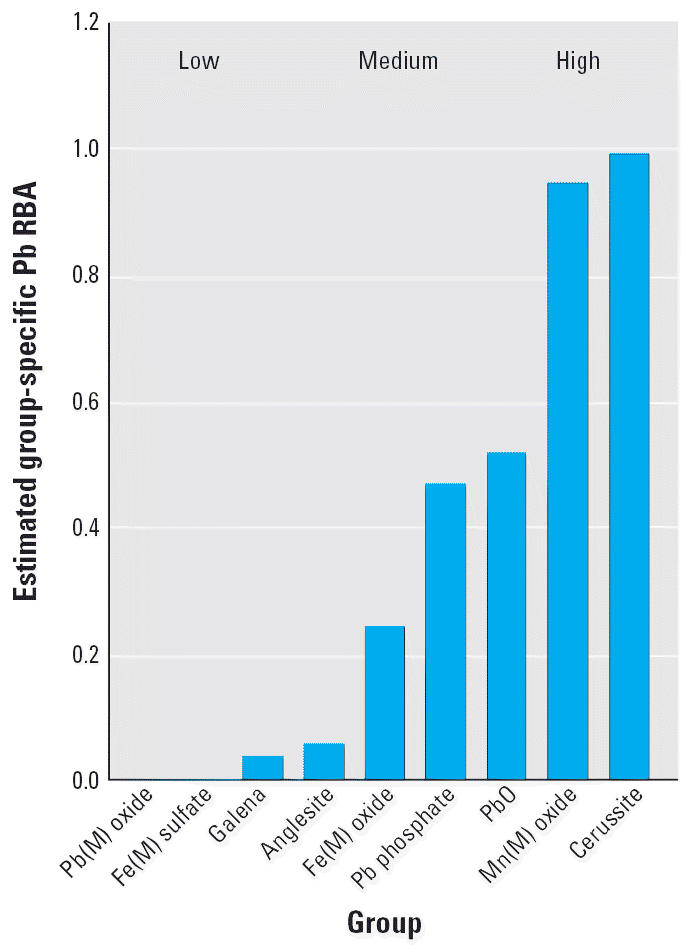
Estimated group-specific RBA values, with groups as defined in Table 7.

The resulting estimates of the group-specific RBA values are shown in Figure 8. There is a wide range of group-specific RBA values. It is important to stress that these group-specific RBA estimates are derived from a very limited data set (nine independent parameter estimates based on only 19 different measurements), so the group-specific RBA estimates are inherently uncertain. In addition, both the measured sample RBA values and the RLM in each phase are subject to additional uncertainty. Therefore, the group-specific RBA estimates should not be considered highly precise and calculation of a quantitative sample-specific RBA value from these estimates is not appropriate. Rather, it is more appropriate to consider the results of this analysis as sufficient to support only semiquantitative (low, medium, high) classification of phase-specific RBA values. As noted above, the estimates apply only to particles that are liberated, not to those that are included.

## Conclusions

Juvenile swine are believed to be a useful model of gastrointestinal absorption in children. The results from the studies conducted during this program indicate that juvenile swine can be used to measure Pb RBA in a variety of soil-like test materials. Each RBA estimate is uncertain because of the variability in response between different animals, but the magnitude of this uncertainty can be quantified to allow risk managers flexibility in choosing a value for use in risk assessment and risk management decision making. If necessary, the magnitude of the uncertainty can be reduced by using more animals per dose group and/or more dose groups to help define the dose–response curves with greater certainty.

Each of the four different end points employed in these studies (blood AUC, liver, kidney, bone) to estimate RBA appear to yield reasonable values, with no one end point being clearly superior to the others. Thus, the best estimate of the RBA value for any particular sample is the average across all four end-point–specific RBA values, and combining results from the independent end points helps increase confidence in the point estimate.

There are clear differences in the RBA of Pb between different types of test material, ranging from near zero to close to 100%. Thus, reliable data on the RBA value for different types of test materials at a site can be very important in improving Pb risk assessments at a site. The U.S. EPA default value for the RBA of Pb in soil is 60% (U.S. EPA 1994a). Of the 17 authentic site soil samples tested in this program, 8 had point estimate values within 20% of the default (i.e., from 40 to 80%), 6 had point estimate RBA values < 40%, and 3 had point estimate values > 80%. Thus, based on this set of samples, the U.S. EPA default value of 60% appears to be a reasonable central tendency value.

Presumably, the RBA value for any one sample is a weighted function of the “phase-specific” RBA values for each Pb phase present in the sample. Available data support the view that certain types of Pb minerals are well absorbed (e.g., cerussite, Mn/Pb oxide), whereas other forms are poorly absorbed (e.g., galena, anglesite). However, the data are not yet sufficient to allow reliable quantitative calculation or prediction of the RBA for a test material based on knowledge of the Pb mineral content alone.

## Figures and Tables

**Table 1:** Description of Phase II test materials.

Test material	Study	Sample designation	Site	Sample description	Pb concentration (ppm)^a^
1	2	Bingham Creek Residential	Kennecott NPL site, Salt Lake City, Utah	Soil composite of samples containing < 2,500 ppm Pb; collected from a residential area (Jordan View Estates) located along Bingham Creek in the community of West Jordan, Utah	1,590
2	2	Bingham Creek Channel Soil	Kennecott NPL site, Salt Lake City, Utah	Soil composite of samples containing ≥ 3,000 ppm Pb; collected from a residential area (Jordan View Estates) located along Bingham Creek in the community of West Jordan, Utah	6,330
3	3	Jasper County High Lead Smelter	Jasper County, Missouri, Superfund site	Soil composite collected from an on-site location	10,800
4	3	Jasper County Low Lead Yard	Jasper County, Missouri, Superfund site	Soil composite collected from an on-site location	4,050
5	4	Murray Smelter Slag	Murray Smelter Superfund site, Murray City, Utah	Composite of samples collected from areas where exposed slag existed on site	11,700
6	4	Jasper County High Lead Mill	Jasper County, Missouri, Superfund site	Soil composite collected from an on-site location	6,940
7	5	Aspen Berm	Smuggler Mountain NPL site, Aspen, Colorado	Composite of samples collected from the racquet club property (including a parking lot and a vacant lot)	14,200
8	5	Aspen Residential	Smuggler Mountain NPL site, Aspen, Colorado	Composite of samples collected from residential properties within the study area	3,870
9	6	Midvale Slag	Midvale Slag NPL site, Midvale, Utah	Composite of samples collected from a water-quenched slag pile in Midvale Slag Operable Unit 2	8,170
10	6	Butte Soil	Silver Bow Creek/Butte Area NPL site, Butte, Montana	Soil composite collected from waste rock dumps in Butte Priority Soils Operable Unit	8,530
11	7	California Gulch Phase I Residential Soil	California Gulch NPL site, Leadville, Colorado	Soil composite collected from residential properties within Leadville	7,510
12	7	California Gulch Fe/Mn PbO	California Gulch NPL site, Leadville, Colorado	Soil composite collected from near the Lake Fork Trailer Park located southwest of Leadville near the Arkansas River	4,320
13	8	California Gulch AV Slag	California Gulch NPL site, Leadville, Colorado	Sample collected from a water-quenched slag pile on the property of the former AV (Arkansas Valley) Smelter, located just west of Leadville	10,600
14	9	Palmerton Location 2	New Jersey Zinc NPL site, Palmerton, Pennsylvania	Soil composite collected on-site	3,230
15	9	Palmerton Location 4	New Jersey Zinc NPL site, Palmerton, Pennsylvania	Soil composite collected on-site	2,150
16	11	Murray Smelter Soil	Murray Smelter Superfund site, Murray City, Utah	Soil composite collected on-site	3,200
17	11	NIST Paint	NA	A mixture of approximately 5.8% NIST SRM 2589^b^ and 94.2% low-Pb soil (< 50 ppm) collected in Leadville, Colorado	8,350
18	12	Galena-Enriched Soil	NA	A mixture of approximately 1.2% galena^c^ and 98.8% low-Pb soil (< 50 ppm) collected in Leadville, Colorado	11,200
19	12	California Gulch Oregon Gulch Tailings	California Gulch NPL site, Leadville, Colorado	A composite of tailings samples collected from the Oregon Gulch tailings impoundment	1,270

Abbreviations: Fe, iron; Mn, manganese; NA, not applicable; NIST, National Institute of Standards and Technology; NPL, National Priorities List; PbO, Pb oxide; SRM, Standard Reference Material.

a Samples were analyzed for Pb by inductively coupled plasma-atomic emission spectrometry in accordance with U.S. EPA Method 200.7 (U.S. EPA. 1994b); all samples were dried and sieved to 250 μm before analysis, except for the two Aspen samples (study 5), which were sieved to 150 μm.

b SRM 2589, composed of paint collected from the interior surfaces of houses in the United States, contains a nominal Pb concentration of 10% (100,000 ppm); the material is powdered, with > 99% of the material < 100 μm in size.

c Galena consisted of a mineralogic (i.e., native) crystal of pure galena that was ground and sieved to obtain fine particles < approximately 65 μm.

**Table 2:** Values for the variance model parameters *k*_1_ and *k*_2_.

End point	*k*_1_	*k*_2_
Blood AUC	−1.3226	1.5516
Liver	−2.6015	2.0999
Kidney	−1.8499	1.9557
Femur	−1.9713	1.656

**Table 3:** RLM (%) of mineral phases observed in test materials.

	Test material																		
Phase	1	2	3	4	5	6	7	8	9	10	11	12	13	14	15	16	17	18	19
Anglesite	—	28	1	0.5	1.0	2	7	1	—	36	10	—	2	6	4	—	1	—	—
As(M) oxide	—	—	—	—	—	—	—	—	—	—	—	—	—	—	—	0.003	—	—	—
Calcite	—	—	0.2	—	—	0.1	—	—	—	—	—	—	—	—	—	—	—	—	—
Cerussite	2	0.3	32	81	1.1	57	62	64	4	0.3	20	—	1	—	—	14	55	—	—
Clay	—	—	0.018	0.003	—	0.017	0.1	—	—	0.1	—	0.01	—	0.03	0.13	—	—	—	—
Fe/Pb oxide	6	3	14	2	2	10	9	7	0.3	7	6	8	51	2	2	0.13	—	—	—
Fe/Pb sulfate	22	30	3	1	0.3	1	5	5	0.1	20	6	3	0.3	1	—	0.6	—	—	—
Galena	—	9	—	8	9	3	12	17	6	12	2	—	3	—	—	20	—	100	100
Pb barite	—	0.04	—	—	—	0.01	0.06	—	—	0.007	0.15	0.14	—	1	0.1	—	—	—	—
Organic Pb	—	0.3	—	—	—	—	0.03	0.03	—	—	0.11	0.11	1	—	—	—	—	—	—
PbO	—	—	0.09	—	69	7	—	—	—	—	—	—	—	—	—	27	44	—	—
Pb phosphate	50	26	21	6	—	7	1	1	—	3.6	30	15	—	24	1	—	—	—	—
Pb silicate	—	—	—	0.04	—	0.5	—	—	—	—	1.9	0.8	—	—	1.4	—	—	—	—
Pb vanadate	—	—	—	—	—	—	—	—	—	—	0.1	0.4	—	—	18	—	—	—	—
Mn/Pb oxide	18	2	2	2	0.8	9	4	5	—	20.2	22	72	—	66	66	—	—	—	—
Native Pb	—	—	22	—	0.7	2	—	—	15	—	—	—	—	—	—	—	—	—	—
Pb(M) oxide	—	—	—	—	4	—	—	—	26	—	—	—	—	—	7	3	—	—	—
Pb/As oxide	2	1	—	0.15	6	—	—	—	33	—	0.1	—	31	—	—	29	—	—	—
PbO/cerussite	—	—	—	—	—	—	—	—	—	—	1	—	—	—	—	—	—	—	—
Slag	—	—	4	—	7	1	—	—	16	—	1	—	10	—	—	6	—	—	—
Sulfosalts	—	—	—	—	—	—	—	—	0.4	—	—	—	—	—	—	—	—	—	—
Zn/Pb silicate	—	—	—	—	0.03	—	—	—	—	—	—	—	—	—	2	—	—	—	—

Abbreviations: —, not observed; As, arsenic; M, metal; Zn, zinc.

**Table 4:** Matrix associations of Pb particles in test materials.

	Particle frequency (%)		RLM (%)	
Test material	Liberated	Included	Liberated	Included
1	100	0	100	0
2	100	0	100	0
3	81	19	76	24
4	100	0	94	6
5	87	13	77	23
6	96	4	93	7
7	86	14	93	8
8	98	2	94	6
9	91	9	77	23
10	91	9	91	9
11	79	21	65	35
12	98	2	100	0
13	78	22	80	20
14	100	0	100	0
15	79	21	89	11
16	80	20	70	30
17	100	0	100	0
18	100	0	100	0
19	2	98	5	95

**Table 5:** Length distributions (%) for Pb-bearing particles in test materials.

	Particle size (μm)								
Test material	< 5	5–9	10–19	20–49	50–99	100–149	150–199	200–249	> 250
1	38	22	19	16	4	2	0	0	0
2	66	13.6	10	6.1	3	1	0	0	0
3	44	19	8	8	9	9	2	1	1
4	29	20	21	20	8	3	0	0	0
5	14	13	15	6	20	24	4	3	0
6	23	21	22	19	9	6	1	1	0
7	27	19	22	17	8	6	1	1	0
8	38	35	12	8	4	2	0	0	0
9	6	1	3	4	20	29	18	13	5
10	23	15	14	23	14	9	2	1	0
11	24	9	18	22	15	9	1	1	1
12	26	19	24	17	10	4	0	0	0
13	19	8	8	5	9	19	10	13	9
14	26	23	25	18	6	1	0	0	0
15	25	15	21	25	13	2	0	0	0
16	23	10	29	17	6	8	3	3	1
17	76	4	6	8	6	0	0	0	0
18	48	2	4	41	4	0	0	0	0
19	85	8	6	0	0	0	0	0	0

**Table 6:** Estimated Pb RBA values for test materials.

	Blood AUC			Liver			Kidney			Femur			Point estimate		
Test material	RBA	LB	UB	RBA	LB	UB	RBA	LB	UB	RBA	LB	UB	RBA	LB	UB
1	0.34	0.23	0.50	0.28	0.20	0.39	0.22	0.15	0.31	0.24	0.19	0.29	0.27	0.17	0.40
2	0.30	0.20	0.45	0.24	0.17	0.34	0.27	0.19	0.37	0.26	0.21	0.31	0.27	0.19	0.36
3	0.65	0.47	0.89	0.56	0.42	0.75	0.58	0.43	0.79	0.65	0.52	0.82	0.61	0.43	0.79
4	0.94	0.66	1.30	1.00	0.75	1.34	0.91	0.68	1.24	0.75	0.60	0.95	0.90	0.63	1.20
5	0.47	0.33	0.67	0.51	0.33	0.88	0.31	0.22	0.46	0.31	0.23	0.41	0.40	0.23	0.64
6	0.84	0.58	1.21	0.86	0.54	1.47	0.70	0.50	1.02	0.89	0.69	1.18	0.82	0.51	1.14
7	0.69	0.54	0.87	0.87	0.58	1.39	0.73	0.46	1.26	0.67	0.51	0.89	0.74	0.48	1.08
8	0.72	0.56	0.91	0.77	0.50	1.21	0.78	0.49	1.33	0.73	0.56	0.97	0.75	0.50	1.04
9	0.21	0.15	0.31	0.13	0.09	0.17	0.12	0.08	0.18	0.11	0.06	0.18	0.14	0.07	0.24
10	0.19	0.14	0.29	0.13	0.09	0.19	0.15	0.09	0.22	0.10	0.04	0.19	0.14	0.06	0.23
11	0.88	0.62	1.34	0.75	0.53	1.12	0.73	0.50	1.12	0.53	0.33	0.93	0.72	0.38	1.07
12	1.16	0.83	1.76	0.99	0.69	1.46	1.25	0.88	1.91	0.80	0.51	1.40	1.05	0.57	1.56
13	0.26	0.19	0.36	0.19	0.11	0.32	0.14	0.08	0.25	0.20	0.13	0.30	0.20	0.09	0.31
14	0.82	0.61	1.05	0.60	0.41	0.91	0.51	0.30	0.91	0.47	0.37	0.60	0.60	0.34	0.93
15	0.62	0.47	0.80	0.53	0.37	0.79	0.41	0.25	0.72	0.40	0.32	0.52	0.49	0.29	0.72
16	0.70	0.54	0.89	0.58	0.42	0.80	0.36	0.25	0.52	0.39	0.31	0.49	0.51	0.29	0.79
17	0.86	0.66	1.09	0.73	0.52	1.03	0.55	0.38	0.78	0.74	0.59	0.93	0.72	0.44	0.98
18	0.01	0.00	0.02	0.02	0.00	0.04	0.01	0.00	0.02	0.01	−0.01	0.03	0.01	0.00	0.03
19	0.07	0.04	0.13	0.11	0.04	0.21	0.05	0.02	0.09	0.01	−0.04	0.06	0.06	−0.01	0.15
14R^a^	0.71	0.55	0.99	1.25	0.82	2.03	0.54	0.35	0.80	0.95	0.69	1.30	0.86	0.43	1.52

Abbreviations: LB, 5% lower confidence bound; UB, 95% upper confidence bound.

a Repeat analysis of test material 14.

**Table 7:** Grouped Pb phases.

Group	Group name	Phase constituents
1	Galena	Galena (PbS)
2	Cerussite	Cerussite
3	Mn(M) oxide	Mn/Pb oxide
4	PbO	PbO
5	Fe(M) oxide	Fe/Pb oxide (including Fe/Pb silicate) Zn/Pb silicate
6	Pb phosphate	Pb phosphate
7	Anglesite	Anglesite
8	Pb(M) oxide	As(M) oxide Pb silicate Pb vanadate Pb(M) oxide Pb/As oxide
9	Fe(M) sulfate	Fe/Pb sulfate Sulfosalts
10	Minor constituents	Calcite Clay Pb barite Organic Pb Native Pb PbO/cerussite Slag

M, metal.
